# Mindfulness in Pregnancy and Postpartum: Protocol of a Pilot Randomized Trial of Virtually Delivered Mindfulness-Based Cognitive Therapy to Promote Well-Being during the Perinatal Period

**DOI:** 10.3390/ijerph21050622

**Published:** 2024-05-14

**Authors:** Shannon D. Donofry, Dayna Winograd, Diva Kothari, Christine C. Call, Kelsey E. Magee, Riley J. Jouppi, Rachel P. Kolko Conlon, Michele D. Levine

**Affiliations:** 1 RAND, 4570 Fifth Avenue, Suite 600, Pittsburgh, PA 15213, USA; 2 Department of Psychology, University of Pittsburgh, Pittsburgh, PA 15260, USA; 3 Department of Psychiatry, University of Pittsburgh School of Medicine, Pittsburgh, PA 15213, USA; 4 Department of Obstetrics, Gynecology, and Reproductive Sciences, University of Pittsburgh, Pittsburgh, PA 15213, USA

**Keywords:** pregnancy, perinatal, postpartum, mindfulness, cognitive behavioral therapy

## Abstract

Background: During the period from pregnancy through the first year postpartum, vulnerable individuals are at elevated risk for the onset or worsening of psychological distress, and accessible (e.g., virtually delivered) mental health interventions are needed. Research suggests that Mindfulness-Based Cognitive Therapy (MBCT) can effectively mitigate psychological distress, although few studies have evaluated MBCT in the perinatal period, and samples have been clinically homogenous. Thus, we have designed and are conducting a pilot trial of virtually delivered MBCT with pregnant individuals experiencing a range of psychological symptoms to assess its feasibility and preliminarily explore its effectiveness. Here, we present the study protocol. Methods: Eligible participants (target *N* = 70) are ≥18 years with pregnancies between 12 and 30 weeks of gestation. Participants complete a diagnostic interview, self-report symptom ratings, and a computerized cognitive battery assessing self-regulation at the baseline. Participants are then randomized to either MBCT or care as usual. The MBCT intervention involves eight weekly group sessions delivered virtually, with each session focusing on a mindfulness practice followed by group discussion and skill development. Participants in the intervention group are also encouraged to practice mindfulness skills between sessions. Participants in the control condition are provided with information about mindfulness and treatment resources. Baseline measures are repeated following the eight-week intervention period and at three months postpartum. Conclusions: This pilot study is designed to evaluate the feasibility of virtually delivered MBCT and explore group differences in psychological symptoms during the perinatal period, and will lay the foundation for a larger clinical trial focused on optimizing this intervention to improve psychological functioning among diverse pregnant individuals.

## 1. Introduction

The perinatal period, defined as pregnancy through the first year postpartum, is a time of increased susceptibility to the onset, worsening, or recurrence of psychiatric symptoms due to the numerous physiological, psychological, social, and emotional changes that occur during this period [1,2,3,4]. Nearly 80% of individuals experience postpartum dysphoria (i.e., mood swings, anxiety, sadness, or related symptoms that last for up to two weeks following delivery), and about 20% of individuals meet clinical criteria for major depression during the perinatal period [1,5,6]. Other psychiatric symptoms that may emerge or worsen during pregnancy and postpartum include anxiety, obsessions, compulsions, and mania [7,8]. Further, individuals from minoritized and under-resourced communities are more likely to experience poor behavioral health outcomes during the perinatal period [5,9,10]. Unfortunately, behavioral health conditions are one of the primary drivers of the maternal morbidity and mortality crisis in the U.S., with nearly a quarter of all maternal deaths being attributable to behavioral health conditions, including deaths by suicide and overdoses [11,12]. These deaths are considered preventable with appropriate treatment [11,12]. Despite the high prevalence of perinatal mental health conditions, the majority of individuals do not receive adequate treatment during pregnancy and postpartum [2,5].

Behavioral and psychological interventions are effective alternatives or adjuncts to medication for treating and preventing perinatal mental health disorders and are the preferred form of treatment among pregnant and postpartum individuals [13]. Mindfulness-Based Cognitive Therapy (MBCT), an eight-week group-based treatment modality designed specifically to prevent recurrence of depressive symptoms in non-pregnant populations [14], may be an effective intervention approach to address perinatal mental health concerns. MBCT combines principles of cognitive behavioral therapy and mindfulness meditation to help individuals change the way in which they relate to automatic negative thinking patterns that trigger symptom onset and/or recurrence. For instance, individuals learn to increase awareness and acceptance of negative thoughts and emotions, which improves tolerance of these experiences and facilitates more effective goal-concordant action. Several recent trials of MBCT adapted for use in a perinatal population have demonstrated that MBCT is effective in preventing or mitigating symptoms of depression and anxiety among pregnant and postpartum individuals [15,16,17,18,19]. High rates of self-reported satisfaction with and engagement in treatment have been documented, which is notable given the unique demands of the perinatal period [15,16,17,18,19,20]. 

Despite these promising findings, the existing literature is limited in two crucial ways. First, previous studies have predominantly focused either on examining the efficacy of MBCT as a preventative treatment in currently healthy pregnant individuals with a history of depression [15,16] or among community samples of pregnant individuals with a relatively limited range and severity of psychological symptoms, typically subthreshold depression or anxiety [18,21,22,23]. Thus, it is unclear whether MBCT is beneficial for individuals experiencing acute symptomatology or for individuals meeting diagnostic criteria for a psychiatric illness during pregnancy. This limitation has made it challenging to extend MBCT treatment access to clinical populations who could potentially benefit from it during this period of heightened vulnerability.

A second key limitation is the lack of research examining the mechanisms through which MBCT improves psychological functioning and overall well-being [24,25], particularly for individuals in the perinatal period. Evidence from basic science suggests that mindfulness training may enhance self-regulatory skills [26,27,28], defined broadly as the ability to volitionally control processes such as thinking, attention, emotion, and behavior in a goal-concordant manner. For example, a brief mindfulness intervention has been shown to acutely improve inhibitory control in a non-clinical sample [29]. Thus, it is possible that MBCT may sharpen self-regulation skills, potentially by engaging the two core components of the practice: attention monitoring and non-judgmental acceptance of present-moment experience [28]. However, few studies have rigorously evaluated possible mechanisms of action of MBCT, and none have done so among pregnant individuals. Evaluating the ways in which group-based MBCT can improve self-regulatory capacity during pregnancy will enhance our understanding of the role of mindfulness-based interventions in promoting behavioral and cognitive change during key life transitions. 

To address these limitations, we are conducting a small pilot randomized controlled trial of MBCT delivered virtually among individuals reporting mild or greater severity distress during pregnancy. The aims of this pilot randomized controlled trial are to (1) determine the feasibility and acceptability of MBCT delivered via telehealth among a community sample of pregnant individuals with a range of clinical presentations, and (2) explore group differences in psychological functioning and self-regulation at three months postpartum to establish the preliminary effectiveness of MBCT for improving these outcomes. Below, we describe the methodological protocol we are implementing to test these aims.

## 2. Methods

**Figure 1:**
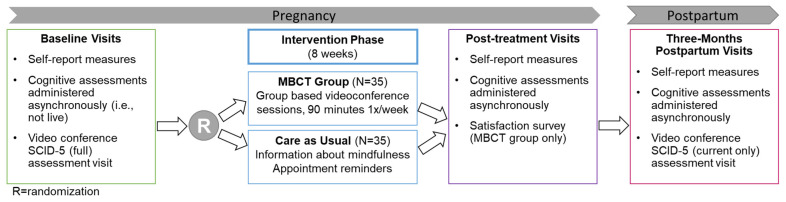
Study timeline. Note that participants were not deemed fully eligible until all baseline assessments were completed.

Overview and study design. The study timeline is depicted in Figure 1. This study was registered on ClinicalTrials.gov (identifier: NCT05137925) and approved by the University of Pittsburgh Institutional Review Board prior to the initiation of any study activities. 

Individuals are enrolled during the second or third trimester of their pregnancies and randomly assigned to the MBCT intervention or a comparison condition involving care as usual (CAU). At baseline, prior to randomization, eligible participants complete (1) a diagnostic interview via a videoconference call to assess their psychiatric history, (2) self-report of sociodemographic information, such as age, racial identity, household income, and marital status, and (3) self-report symptom questionnaires and cognitive assessments via a secure online survey portal. Enrollment occurs in cohorts, with cohort-specific enrollment proceeding until at least five participants have been randomized to the intervention group to ensure a sufficient group size. With the exception of the diagnostic interview, baseline measures are repeated immediately following the eight-week intervention period. Participants then complete all measures, including a diagnostic interview, at a three months-postpartum follow-up visit. Participants are compensated for each assessment they complete. Recruitment for the trial began in April 2022 and is ongoing.

Community engagement and recruitment. Several recruitment methods are being employed for this study. First, to ensure we are reaching and enrolling populations for whom there is the greatest need of services, we are employing a community-engaged research approach to invite key community stakeholders to provide input on the research process. We are convening 1–2 community advisory board meetings comprising individuals with lived experience of managing psychological distress during pregnancy and postpartum, as well as providers who deliver perinatal health and mental health services, such as doulas and therapists. The advisory board members will share their perspectives and advise on key decisions and approaches, particularly as they pertains to recruiting and retaining individuals from minoritized backgrounds in the trial given that these individuals experience the highest rates of maternal morbidity and mortality and are often not included in mindfulness-based intervention research [30,31]. In addition, we will meet with administrative and clinical personnel at community-based obstetrics clinics who primarily serve patients from under-resourced and minoritized communities to share information about the trial and develop informal partnerships to facilitate recruitment from their patient populations. We will also attend community resource events to build relationships with organizations focused on providing services and support to individuals during the perinatal period and to share information about our research. Finally, we will volunteer our time and expertise at community events, including by offering to lead psychoeducational and mindfulness sessions.

Other recruitment efforts include an online research registry managed by the University of Pittsburgh that connects interested community members with ongoing research opportunities, as well as the distribution of fliers throughout local communities, with a particular focus on organizations and businesses that are frequented by individuals in the perinatal period or their loved ones. Participants are also recruited via ClinicalTrials.gov and referrals from professional (e.g., obstetrics providers) and non-professional (e.g., friends, partners) contacts.

Eligibility screening. Eligible participants are ≥18 years, currently pregnant with pregnancies between 12 and 30 weeks of gestation and endorse mild or greater severity psychological distress (total scores of ≥5 on the Edinburgh Postnatal Depression Scale (EPDS) and/or the Perceived Stress Scale (PSS)). Because our aim was to recruit a sample of individuals with a wide range of presenting symptoms and symptom severity levels, we did not want to limit inclusion to only those individuals with high-severity symptoms or to those meeting diagnostic criteria for a psychiatric condition. On the other hand, given that one of the aims of the project was to evaluate MBCT among pregnant individuals experiencing distress, we chose to exclude individuals who reported no distress as this would limit our ability to demonstrate an effect of treatment. Our rationale for selecting ≥5 as the score cut off for inclusion in these measures, therefore, was to balance between these two extremes, and to ensure that our sample reflected the range of symptoms and levels of severity that are observed among pregnant individuals in community settings (i.e., symptoms ranging from mild to severe). Exclusion criteria include (1) current suicidal or homicidal ideation as assessed verbally during screening, (2) current psychosis, (3) untreated mania, (4) active substance use disorder, and/or (5) lack of access to high-speed internet or cellular networks.

Advertisements for the study inform participants that the goal of the research is to evaluate whether or not mindfulness skills help improve well-being during pregnancy and include a QR code linking interested individuals to a secure online screening survey. Potential participants are initially screened via this survey to determine preliminary eligibility and continued interest in the study. If deemed eligible based on this screening, potential participants undergo a video-conferencing call via a version of the Zoom software version 6.0.0 [32] that is compliant with security standards outlined in the U.S. Health Insurance Portability and Accountability Act (HIPAA) [33] to determine their full eligibility to continue in the study. During this call, informed consent is acquired through Research Electronic Data Capture (REDCap), which is a secure web-based platform operated by the Clinical and Translational Science Institute at the University of Pittsburgh. Consent is securely documented electronically, and participants are emailed a copy of the signed consent form to keep for their records. Once consent is obtained, participants complete an assessment that includes the EPDS and PSS as well as the Structured Clinical Interview for DSM-5-Research Version (SCID-5-RV). If participants are deemed fully eligible based on the responses provided during this assessment, they then complete a larger battery of self-report measures and computerized cognitive tasks via REDCap. The timepoints of the assessments and estimated duration of each assessment are reported in Table 1, and the study timeline is depicted in Figure 1. Following this procedure, eligible participants are randomized to their respective groups.

Randomization. Participants are randomized with equal probability of being randomized to the MBCT intervention or CAU group. Prior to the start of the trial, a randomization list was generated using a random allocation function with the randomizeR package, version 2.0 [34], in R version 4.0.3 [35].

### 2.1. Self-Report and Interview-Based Measures

Depressive symptoms. The presence and severity of depressive symptoms are measured using the 10-item Edinburgh Postnatal Depression Scale (EPDS; [36,37]), which is a widely used instrument validated in pregnancy and the postpartum period [38,39]. Participants rate the severity of depressive symptoms experienced in the past week on a Likert scale ranging from 0 to 3. Scores are summed up to form a total score, with higher scores reflecting more severe symptomatology. The EPDS has demonstrated adequate internal consistency in prior research (Cronbach’s α = 0.77; [37]).

Perceived stress. Perceptions of daily life stress are measured using the Perceived Stress Scale (PSS; [40]). This 10-item questionnaire asks participants to rate items on a 5-point Likert scale from 0 (never) to 4 (very often) on how overwhelming, unpredictable, or uncontrollable they find their lives. Responses are summed up to form a total score, with higher scores indicating greater perceived stress. This scale has been shown to exhibit satisfactory reliability (Cronbach’s α = 0.85) and validity [41], including during the perinatal period [42].

Generalized worry. Symptoms of generalized worry are assessed using the 16-item Penn State Worry Questionnaire (PSWQ; [43,44]). Participants rate each item on a 5-point Likert scale from 1 (not at all typical of me) to 5 (very typical of me). Higher scores are indicative of more severe worry. The instrument has satisfactory internal consistency and construct validity [45], including among individuals evaluated during pregnancy or the postpartum period [46].

Rumination. Participants complete the Ruminative Responses Scale (RRS; [47]), a 22-item scale assessing the tendency to engage in a negative, self-focused thinking style in response to negative experiences. Participants rate each item on a 4-point Likert scale ranging from 1 (never) to 4 (always). A higher score indicates a higher degree of ruminative symptoms. The RRS has been shown to possess adequate internal consistency and convergent validity [48,49], including during the perinatal period [50].

Emotion regulation. The 10-item Emotion Regulation Questionnaire (ERQ; [51]) is administered to evaluate the influence of the intervention on use of common emotion regulation strategies (e.g., suppression and reappraisal). Participants answer each item on a 7-point Likert scale ranging from 1 (strongly disagree) to 7 (strongly agree). For the reappraisal and suppression subscales, mean scores across the respective subscale items are calculated, with higher mean scores indicating more frequent usage of each emotion regulation strategy. The ERQ has been shown to demonstrate adequate internal consistency and convergent validity [52,53] and has been used to evaluate emotion regulation during the perinatal period [54,55,56,57].

Mindfulness. To evaluate whether the intervention is associated with increased use of mindfulness in daily life, the Five Facet Mindfulness Questionnaire (FFMQ; [58,59]) is administered. It is a 39-item survey that queries participants on five skills associated with regular mindfulness practice: (1) observing, (2) describing, (3) acting with awareness, (4) non-reactivity, and (5) non-judging. Participants answer each item on a 5-point Likert scale, ranging from 1 (never or rarely true) to 5 (very often or always true). The FFMQ has shown satisfactory construct validity and reliability [60,61], including during the perinatal period [62,63].

Psychiatric diagnoses. Current and past psychiatric diagnoses are evaluated using the Structured Clinical Interview for DSM-5 Disorders-Research Version (SCID-5-RV; [64]). The full SCID-5-RV is administered at baseline to assess both lifetime and current diagnostic status, while only the current modules are administered at three months postpartum (see Figure 1). These interviews are conducted by trained assessors with at least a bachelor’s degree. Training includes review of didactic materials and videos, and scoring of pre-recorded diagnostic interviews that have previously been rated by master’s-level assessors. Prior to conducting study assessments, assessors must achieve ≥90% agreement with the diagnostic ratings of the master’s-level assessors. The final stage of training involves the staff person conducting diagnostic interviews with two participants while being observed by a master’s-level assessor, after which feedback is provided to address areas for improvement. Diagnoses are confirmed via bi-weekly consensus discussions among the research team, which includes four licensed clinical psychologists. The SCID-5 has been validated for remote administration and used in other virtual behavioral intervention trials [65,66,67,68].

### 2.2. Cognitive Assessments Administered to Assess Self-Regulation

Cognitive assessments are administered remotely using the Inquisit platform by Millisecond [69]. Participants are provided the link to access the platform once they complete the REDCap surveys and are not supervised while they complete their cognitive assessments. Participants are given detailed instructions regarding how to complete the cognitive assessments prior to starting and are provided with contact information to reach research staff if they encounter errors or have questions. Research staff monitor incoming data from the Inquisit platform to track participant completion of the tasks. Inquisit Millisecond has been widely used for online administration of neuropsychological tests (e.g., [70,71,72,73,74,75,76,77,78,79]).

Go/No-Go Task (GNG). The computerized GNG task requires participants to monitor series of stimuli presented in the center of a computer screen and respond as rapidly as possible by pressing a mouse button to respond to target stimuli (Go cues), while withholding a response to non-target stimuli (No-Go cues) [80]. The frequency of Go cues creates a prepotent tendency to respond that must then be inhibited for No-Go cues, which provides a measure of the ability to inhibit a prepotent response [81], with lower scores indicating a greater tendency towards disinhibited behavior. We are currently using this version of the Go/No-Go task in another behavioral trial with individuals in the perinatal period [82].

Stroop Color Word Test. The Stroop Color Word Test is a computerized task in which participants must name the color of a written color word while inhibiting the impulse to read it. The difference between mean response times of correct responses on incongruent and control trials is used to compute an interference score, which provides a measure of attention, with lower scores indicating greater attentional deficits and poorer response inhibition [83]. The Stroop test has been used to assess cognitive functioning in pregnant populations [84,85,86]. 

Wisconsin Card Sorting Task (WCST). The WCST is a computerized task in which participants must match a target card with one of four category cards under changing conditions. The number of incorrect responses that would have been correct for the preceding condition provides a measure of cognitive inflexibility, with higher scores indicating greater cognitive inflexibility [87]. The WCST has been used to evaluate executive functioning in pregnancy [88].

Delay Discounting Task (DDT). The DDT is a computerized assessment of impulsive decision-making in which participants are presented with a series of monetary values available either immediately or after a temporal delay of varying lengths [89]. Indifference points reflecting the time at which each individual switches from choosing the immediately available value to the delayed value are calculated for each delay interval as the midpoint between the lowest immediate value chosen and the rejected next lowest immediate value [90]. These indifference points are then fit to a hyperbolic function according to the following formula: V = A/(1 + kD), where, V is the value of the indifference point, A is the USD 100 delayed outcome, D is the length of the delay, and k is the magnitude from low to high of the degree to which an individual discounted delayed outcomes [91,92,93]. The DDT has been used to assess impulsivity during the perinatal period [94,95].

### 2.3. Intervention Conditions

Mindfulness-Based Cognitive Therapy (MBCT). The eight-week, group-based MBCT intervention is modeled after the format developed by Dimidjian et al., 2015 and 2016 [15,16], which included adaptations of the standard MBCT protocol to fit the needs of individuals in the perinatal period_._ We additionally made the following adaptations aimed at increasing the accessibility and acceptability of the intervention for pregnant individuals: (1) virtual delivery of the intervention via HIPAA-compliant Zoom, (2) a reduction in the session length from 120 min to 90 min, and (3) emphasis on informal and brief meditation, including formal meditation practices of a shorter duration. Each MBCT session is led by a doctoral-level clinician with training in MBCT and focuses on varying themes (e.g., practicing loving kindness toward the self, learning to set boundaries, and building social support), which are described in more detail in Table 2. Participant engagement is measured through study attendance and the completion of weekly tasks and homework logs. During the intervention period, participants are asked to complete a mindfulness practice five days per week to support the building of a routine, habitual practice outside of the group. Participants record the duration, type of meditation, and other findings that arose during their self-guided practice on homework logs. Participants also complete other homework tasks related to the theme presented during the session in a given week, such as the monitoring of pleasant events and seeking support from loved ones. Participant satisfaction is gauged using a brief qualitative survey completed after the interventional period during which participants discuss their experience openly. Responses are recorded to conduct a thematic analysis. 

Care as Usual (CAU). Participants who are randomized to the CAU group are provided with information regarding the benefits of mindfulness in pregnancy and are offered referrals for psychotherapy within the community. Additionally, study team members maintain email or phone contact with participants between assessment periods. As with the MBCT group, baseline measures are repeated immediately after the eight-week intervention period and at three months postpartum (see Figure 1).

Participant attrition. RCTs of mindfulness-based interventions in pregnancy indicate ~15–20% attrition [15,16,17,19,96]. To minimize attrition in the current trial, participants are compensated for the completion of assessments, and the amount of compensation increases by USD 10 at each successive timepoint. Participants do not complete all assessments in a single session, and the majority of assessments are completed asynchronously. Compensation is provided for each assessment type participants complete, meaning that payments are proportional to the number of assessments completed rather than being given only for 100% completion. Specifically, participants are eligible to earn compensation (1) after the completion of self-report measures, (2) after the completion of cognitive measures, and (3) after the completion of the diagnostic interview. Further, participants are included in a drawing for an additional USD 50 if they complete the postpartum assessments within two weeks of being contacted to. Participants randomized to the MBCT group are further eligible for an attendance bonus of up to USD 20 if they attend five or more sessions. In addition to incentivizing participation through a graded compensation strategy, research staff are also in regular contact with participants throughout the study period, including with individuals who are randomized to the CAU group. The mode of contact with participants is based on participants’ expressed preferences and availability, and emphasizes asynchronous forms of communication such as text messaging and email. Further, participants self-schedule assessment appointments using a calendar application synced with the research staff’s availability, reducing the need for communication between staff and participants about scheduling. Finally, post-intervention and postpartum follow-up assessments are scheduled at baseline in collaboration with the participants based on their anticipated availability so that the time is reserved well in advance without the need for additional back-and-forth communication to identify a convenient time. Research staff then send reminders in the weeks and days leading up to the appointment via participants’ preferred form of communication.

Planned statistical analyses. Prior to hypothesis testing, data will be examined to confirm that the data structure meets analytic assumptions. Aim 1 will be evaluated first by examining the number of individuals who complete screening, calculating the recruitment success rate (the number of participants enrolled divided by the number of those deemed eligible), and evaluating the primary drivers of participant ineligibility by calculating the percentage of those whose participation was discontinued by each reason documented. We will also evaluate retention rates. For the MBCT group, successful retention will be defined in two ways: (1) attending at least four of eight sessions offered, consistent with prior research examining MBCT, including during pregnancy [14,16,17,18], and (2) completing post-intervention assessments. For CAU, successful retention will be operationalized as the completion of post-intervention assessments. Previous mindfulness-based intervention studies conducted during pregnancy have demonstrated >80% retention, so our goal is to match or exceed that (e.g., [16,18,25]). Aim 2 will also be evaluated in several ways. First, baseline to post-intervention change scores will be computed for each measure, and descriptive statistics for each change score will be performed. Data from the SCID will be used to determine rates of psychiatric diagnoses at baseline and three months postpartum, and variables will be constructed to indicate whether individuals with diagnoses identified at baseline continue to meet diagnostic criteria at three months postpartum. To formally explore the effect of the intervention on psychological functioning at three months postpartum, within-subjects repeated measures MANOVAs will be performed with EPDS, PSS, PSWQ, and RRS scores as dependent variables. A logistic regression analysis will also be performed to determine the impact of the intervention on diagnostic status at three months postpartum. To evaluate the preliminary effects on self-regulation at three months postpartum, cognitive scores will first be transformed to z-scores and the average z-score will be calculated to derive a composite self-regulation score at each time point. A within-subjects repeated measures MANOVA will be performed with cognitive z-scores as the dependent variable.

Sample size estimation. Because this is a pilot investigation focused on outcomes that have been under-investigated in previous trials of MBCT during the perinatal period, our primary focus is demonstrating feasibility and acceptability, and all analyses conducted to evaluate he effects of MBCT on psychological and cognitive outcomes are therefore considered preliminary. Nevertheless, we include an estimation of the sample size needed based on the power to detect differences between MBCT and CAU in depressive symptoms following the eight-week treatment period. We selected depressive symptoms as the outcome to use for sample size estimation because, compared with other psychological outcomes of interest measured in this study, the impact of MBCT on depressive symptoms is well-established. The estimated effect size is 0.71 based on the results of a recently published meta-analysis of randomized controlled trials of MBCT among individuals with current depressive symptoms [97]. Assuming an 80% power and α of 0.05, we determined that a sample size of 72 (36 per group) would be necessary to detect a treatment effect on continuously measured depressive symptoms. Therefore, our planned sample of 70 individuals would be adequate to evaluate this effect. However, given the likely attrition rate of ~20%, we will also examine confidence intervals and effect sizes when evaluating the impact of MBCT on psychological and cognitive outcomes [98].

## 3. Discussion

Individuals who are pregnant or in the postpartum period are at elevated risk for the onset and worsening of psychiatric symptoms and psychological distress. MBCT, which employs a combination of mindfulness-based skills and cognitive behavioral therapy to address distress-provoking negative thinking patterns, shows promise as an effective treatment and prevention approach among individuals at risk for the emergence or worsening of psychiatric illness during the perinatal period. However, prior research evaluating the efficacy of MBCT in this population has been limited by a primary focus on individuals with remitted depression or low levels of psychological distress and a relative lack of emphasis on understanding mechanisms underlying the effects of MBCT in perinatal populations. To address these limitations, our ongoing trial features several notable adaptations. First, studies that have implemented MBCT have predominantly delivered the intervention via in-person groups and have required a substantial time commitment between the groups themselves and home practice. To expand reach, we are testing virtual sessions that eliminate treatment barriers associated with time commitment, travel, and childcare needs. We also tailored the content to the unique needs of individuals who are pregnant. In addition, the session length was reduced to 90 min and emphasis is placed on mindfulness practices of shorter durations to make it easier for participants to integrate them into their daily lives despite many competing demands, particularly in the context of preparing for and then having a new child. Second, we are also recruiting individuals with a broad range of clinical presentations at varying levels of severity, which will enable an initial evaluation of whether MBCT is effective at reducing distress and improving skill usage in a clinically heterogenous sample of individuals. Third, given the relative dearth of research evaluating self-regulation during pregnancy and postpartum, this study will provide a preliminary indication of whether and how self-regulation changes over the course of the perinatal period. Finally, this study will advance the current understanding of the benefits of MBCT during pregnancy by exploring the effect of MBCT relative to that of CAU on changes in self-regulation. These analyses will set the stage for a larger, fully powered trial to evaluate self-regulation as a potential mechanistic pathway through which MBCT might exert beneficial effects, knowledge of which is currently lacking. This will enable the refinement and optimization of this treatment approach to treat psychological distress during the perinatal period. 

The present study is also extending prior research by incorporating community stakeholder perspectives and adopting a deliberative approach to recruiting a diverse sample of individuals during the perinatal period. Previous studies examining the effectiveness of mindfulness-based interventions for psychological distress during the perinatal period have been limited by a lack of representation of individuals from under-resourced and minoritized communities [99]. We aim to address this limitation by emphasizing engagement with community stakeholders in order to build trust and ensure that our approach will help to meet the mental health needs of individuals during the perinatal period. We are developing partnerships with organizations that provide services to individuals from these communities during the perinatal period, and, through these partnerships, aim to improve our recruitment and retention of individuals who are often under-represented in research and face significant barriers to receiving adequate mental health services [30]. These efforts will primarily focus on local organizations in Western Pennsylvania to prioritize the needs of individuals in the communities where the research team is based. In addition, we will take advantage of the fact that our fully virtual approach to assessment and intervention enables us to recruit nationally and forge connections with national organizations focused on addressing perinatal mental health and health inequities.

With the implementation of the adaptations aimed at enhancing recruitment described above, the completed trial will represent an important initial step toward overcoming limitations of previous research and will serve as a promising basis for a larger, more robust clinical trial testing MBCT and its mechanisms during the perinatal period. Additional research is also planned to evaluate the impact of MBCT on health outcomes during the perinatal period, given evidence that psychological distress during pregnancy may increase the risk for poor maternal physical health [100,101].

## Figures and Tables

**Table 1:** Participant assessments and administration schedule.

Task/Assessment	Collection Period		
	Baseline	Post-Treatment	Three Months Postpartum
Psychological Functioning (Approx. time = 30 min)			
Edinburgh Postnatal Depression Scale	X	X	X
Perceived Stress Scale	X	X	X
Penn State Worry Questionnaire	X	X	X
Ruminative Responses Questionnaire	X	X	X
Five Facets Mindfulness Questionnaire	X	X	X
Psychiatric Diagnoses (Approx. time = 45 min)			
SCID-5—full	X		
SCID-5—current only			X
Self-Regulation (Approx. time = 45 min)			
Go/No-Go Task	X	X	X
Stroop Color-Word Task	X	X	X
Wisconsin Card Sorting Task	X	X	X
Delay Discounting Task	X	X	X
Emotion Regulation Questionnaire	X	X	X
Other (Approx. time = 20 min)			
Demographics (e.g., age, racial identity, household income)	X		
Mindfulness Homework Completion Logs—MBCT only		X	
Satisfaction Survey—MBCT only		X	

Note. MBCT = Mindfulness-Based Cognitive Therapy; SCID = Structured Clinical Interview for DSM-5. Note that the present study employs the research version of the SCID.

**Table 2:** Session content and flow.

Session	Topic	In-Session Targets	Home Practice
1	Doing vs. being mode	Establishing group context and rapport Introducing mindfulness concepts and how they apply to the perinatal period Body scan practice and discussion	Body scan Daily informal mindfulness practice (being mindful while eating, brushing teeth, etc.) Developing awareness of being in the “doing mode” Identifying support people
2	Responding to barriers	Skillfully responding to common challenges that pregnant and postpartum individuals experience when incorporating mindfulness practice into busy daily lives and caregiving Understanding link between thoughts and emotions, and how the “doing mode” contributes to autopilot Body scan practice and discussion	Body scan Brief breath focused meditation Daily informal mindfulness practice Pleasant events monitoring Connecting with support people
3	Mindful breathing and movement	Continued problem solving around engaging in regular mindfulness practice Continued strengthening of distress tolerance in context of mindfulness practice Expanding the daily informal practice to include the developing baby Mindful breathing and mindful movement practice and discussion	Brief breath focused meditation Mindful movement practice Daily informal mindfulness practice Unpleasant event monitoring Connecting with support people
4	Opening to difficulty and uncertainty	Increasing awareness of thoughts, emotions, and sensations rather than engaging in automatic patterns of thinking Increased understanding and awareness of the signs and symptoms of depression and anxiety during pregnancy and common “depression thoughts” and “pregnancy worries”. Continued strengthening of distress tolerance in context of mindfulness practice	Brief breath-focused meditation Mindful movement practice Daily informal mindfulness practice Connecting with support people
5	Thoughts are not facts	Practicing decentering from thoughts and emotions (i.e., acknowledging that these are mental events, not facts) Recognizing recurring thought patterns, particularly negative self-judgments, and ones that strongly hook the attention Continued strengthening of distress tolerance in context of mindfulness practice	Brief breath-focused meditation, especially in difficult moments Unguided meditation practice Daily informal mindfulness practice Connecting with support people
6	Self-care and barriers	Importance of self-care for maintaining well-being Identifying and problem-solving barriers to engaging in self-care commonly experienced by individuals during the perinatal period Practicing self-compassion Loving kindness meditation and discussion	Loving kindness mediation Brief breath-focused meditation, especially in difficult moments Practice self-care and monitor impact on mood and thinking Daily informal mindfulness practice Connecting with support people
7	Expanding support network	Importance of social support for maintaining well-being Identifying and problem-solving barriers to asking others for support, including negative thoughts and emotions Introducing the concept of “relapse signatures”	Brief breath-focused meditation, especially in difficult moments Other formal meditation practice of choice Daily informal mindfulness practice Asking for support from loved ones at least once Developing a personal relapse signature Connecting with support people
8	Relapse prevention	Reviewing relapse signatures and consolidating relapse prevention plans Body scan meditation and discussion Reflections and plans to maintain practice moving forward	
